# Brain insulin signaling suppresses lipolysis in the absence of peripheral insulin receptors and requires the MAPK pathway

**DOI:** 10.1016/j.molmet.2023.101723

**Published:** 2023-04-24

**Authors:** Matthäus Metz, James O'Hare, Bob Cheng, Michelle Puchowicz, Christoph Buettner, Thomas Scherer

**Affiliations:** 1 Division of Endocrinology and Metabolism, Department of Medicine III, Medical University of Vienna, Vienna, 1090 Austria; 2 Department of Medicine, Diabetes, Obesity and Metabolism Institute, Icahn School of Medicine at Mount Sinai, New York, NY, 10029, USA; 3 Department of Nutrition, Case Western Reserve University, Cleveland, OH, 44106, USA; 4 Department of Medicine, Rutgers University, New Brunswick, NJ, 08901, USA

**Keywords:** Brain insulin signaling, Lipolysis, Hypothalamus, MAPK, Mitogen-activated protein kinase, ERK, Extracellular-signaling regulated kinase

## Abstract

**Objectives:**

Insulin's ability to counterbalance catecholamine-induced lipolysis defines insulin action in adipose tissue. Insulin suppresses lipolysis directly at the level of the adipocyte and indirectly through signaling in the brain. Here, we further characterized the role of brain insulin signaling in regulating lipolysis and defined the intracellular insulin signaling pathway required for brain insulin to suppress lipolysis.

**Methods:**

We used hyperinsulinemic clamp studies coupled with tracer dilution techniques to assess insulin's ability to suppress lipolysis in two different mouse models with inducible insulin receptor depletion in all tissues (IR^ΔWB^) or restricted to peripheral tissues excluding the brain (IR^ΔPER^). To identify the underlying signaling pathway required for brain insulin to inhibit lipolysis, we continuously infused insulin +/− a PI3K or MAPK inhibitor into the mediobasal hypothalamus of male Sprague Dawley rats and assessed lipolysis during clamps.

**Results:**

Genetic insulin receptor deletion induced marked hyperglycemia and insulin resistance in both IR^ΔPER^ and IR^ΔWB^ mice. However, the ability of insulin to suppress lipolysis was largely preserved in IR^ΔPER^, but completely obliterated in IR^ΔWB^ mice indicating that insulin is still able to suppress lipolysis as long as brain insulin receptors are present. Blocking the MAPK, but not the PI3K pathway impaired the inhibition of lipolysis by brain insulin signaling.

**Conclusion:**

Brain insulin is required for insulin to suppress adipose tissue lipolysis and depends on intact hypothalamic MAPK signaling.

## Highlights

• Insulin signaling in the brain is sufficient for suppression of lipolysis.
• Hypothalamic MAPK is required for insulin's anti-lipolytic actions.
• MAPK inhibition impairs suppression of lipolysis and glucose production.

## Graphical abstract

**Figure:**
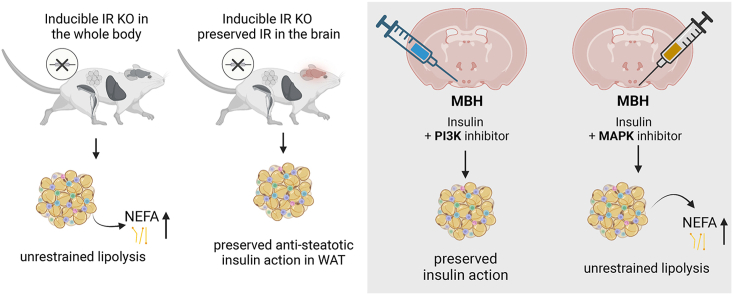

## 1 Introduction

Insulin's ability to antagonize catecholamine-induced lipolysis and to promote lipid storage in the postprandial state is the most important insulin action in white adipose tissue (WAT). Unrestrained lipolysis as a consequence of impaired WAT insulin action is considered a primary cause of systemic insulin resistance [1]. The increased flux of non-esterified fatty acids (NEFA) and glycerol from WAT to the liver fuels hepatic glucose production (hGP) leading to hyperglycemia and compensatory hyperinsulinemia [[2], [3], [4]]. The high lipid supply to the liver also drives hepatic steatosis, which is partially compensated for by increased hepatic triglyceride export contributing to dyslipidemia in insulin resistant individuals [5]. Furthermore, ectopic lipid storage in the liver and the skeletal muscle due to unrestrained lipolysis impairs intracellular insulin signaling [6]. Hence, WAT insulin action is of fundamental importance for systemic glucose and lipid metabolism.

During fasting, norepinephrine released from sympathetic nerve endings stimulates lipolysis by activating β-adrenergic receptors on the plasma membrane of adipocytes increasing cAMP production and consequently cAMP-dependent protein kinase (PKA) activity. PKA phosphorylates the lipid-droplet associated protein perilipin and hormone sensitive lipase (HSL), key components of the lipolytic machinery that are also sensitive indicators of adrenergic signaling [[7], [8], [9]]. Insulin antagonizes β-adrenergic signaling by activating phosphodiesterase isoforms thereby reducing cAMP levels and PKA activity, although other, yet unidentified mechanisms downstream of PKA also contribute to the anti-lipolytic action of insulin [9,10]. Furthermore, insulin stimulates fatty acid uptake and *de novo* lipogenesis in adipocytes and WAT, thereby serving as a pivotal anabolic hormone for lipid storage in WAT [[11], [12], [13]]. While insulin's ability to suppress lipolysis, induce *de novo* lipogenesis and to increase fatty acid uptake are classically explained through the direct and cell autonomous effects of insulin signaling in adipocytes, insulin signaling in the brain exerts complementary effects. Findings from genetic insulin receptor (IR) knockout models illustrate the importance of brain insulin signaling for WAT function. Inducible IR deletion in all tissues results in a severe lipodystrophic phenotype, while IR deletion in all peripheral tissues sparing the brain, or a conditional knock-out of IR in WAT causes only a mild reduction in adipose tissue mass [14,15]. Our previous studies suggest that unrestrained lipolysis underlies this rapidly progressing lipodystrophic phenotype of mice lacking the IR in all tissue including the brain. Insulin infusion into the mediobasal hypothalamus (MBH) suppresses WAT lipolysis independent of peripheral insulin action at the level of the adipocyte. These anti-lipolytic effects of brain insulin signaling are most likely mediated by a suppression of sympathetic outflow to WAT, as evidenced by a reduction of HSL and perilipin phosphorylation [16]. Conversely, blocking endogenous brain insulin signaling by (a) infusing an IR antagonist into the MBH or (b) by using a genetic loss-of-function model in which the IR is deleted throughout the CNS increased WAT lipolysis [16]. In line with the hypothesis that the lipolytic flux from WAT to the liver is a key regulator of hGP [3,4,17], suppression of WAT lipolysis induced by MBH insulin infusion tightly correlates with hGP [16]. Within the hypothalamus, IRs on POMC neurons are required for insulin to suppress WAT lipolysis [18], consistent with the notion that POMC neurons partake in sympathetic outflow pathways [19]. On the cellular level, insulin signals through the phosphatidylinositol 3 kinase (PI3K) and the mitogen activated protein kinase (MAPK) pathways [20,21]. Hypothalamic PI3K signaling has been identified to be required for brain insulin signaling to suppress hGP [22]. However, the signaling pathway that mediates the anti-lipolytic effects of brain insulin remains to be delineated.

There are several methodological limitations of our previous studies exploring brain insulin's role in regulating WAT lipolysis. First, while the stereotaxic insulin infusion studies were designed to achieve hypothalamic insulin concentrations that mimic the postprandial state, infusing insulin intracerebroventricularly (ICV) or intrahypothalamically is a non-physiological route of administration and supraphysiological insulin concentrations in some hypothalamic regions cannot be ruled out [23]. Moreover, these selective intrahypothalamic insulin injection experiments do not account for insulin action in other brain regions that are potentially part of a distributed neuronal network in which the hypothalamus likely plays a key, but not exclusive role. Second, the life-long absence of brain IRs is potentially confounded by developmental adaptations, for example alterations in neuronal networks governing WAT lipolysis [24].

Hence, the objective of this study was to explore the relative importance of brain insulin signaling utilizing an inducible IR knockout in adult animals to avoid developmental compensation and to identify the downstream signaling pathway that mediates the anti-lipolytic effects of brain insulin. We demonstrate that insulin's anti-lipolytic effects are largely preserved in mice with inducible IR deficiency in peripheral tissues, as long as brain IRs are expressed. Furthermore, we identify the MAPK signaling pathway as required for the anti-lipolytic actions of hypothalamic insulin signaling.

## 2 Results

### 2.1 Insulin fails to suppress WAT lipolysis in the absence of brain insulin receptors in male mice

**Figure 1:**
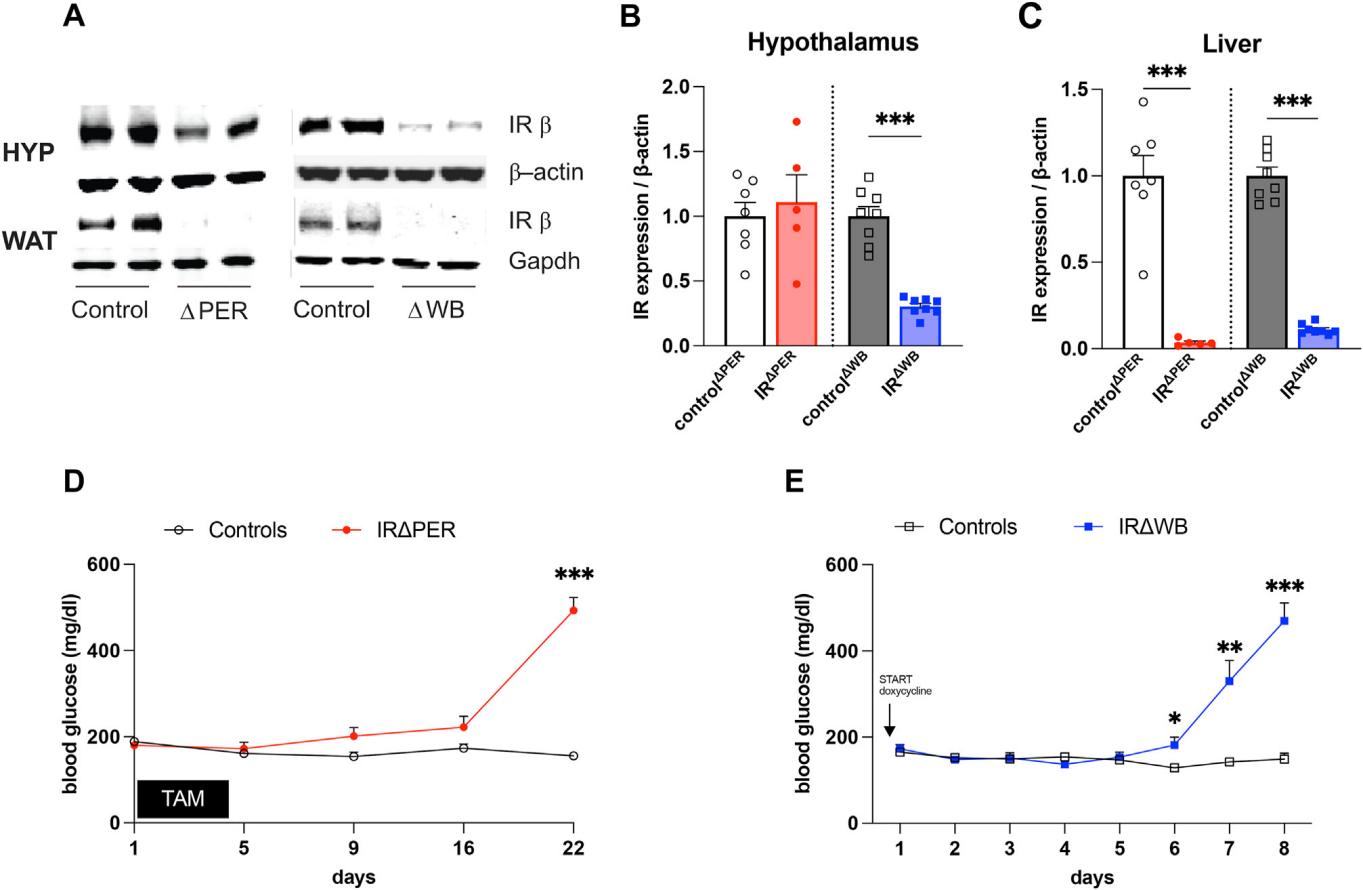
**Induction of insulin receptor deficiency in peripheral tissues or in the whole organism results in hyperglycemia**. (**A**) Confirmatory western blot of IR expression in the brain and WAT of IR^ΔPER^, IR^ΔWB^ mice and respective controls. (**B–C**) Western Blot quantification of IR protein expression in hypothalami (**B**) and livers (**C**) of IR^ΔPER^, IR^ΔWB^ mice and respective controls. (**D**) Blood glucose levels of IR^ΔPER^ mice and controls. (**E**) Blood glucose levels of IR^ΔWB^ and C57BL/6NTac mice (controls^ΔWB^). Data are shown as mean ± SEM. n ≥ 5. ∗p < 0.05, ∗∗p < 0.01 ∗∗∗p < 0.001 by Student's unpaired t test.

**Figure 2:**
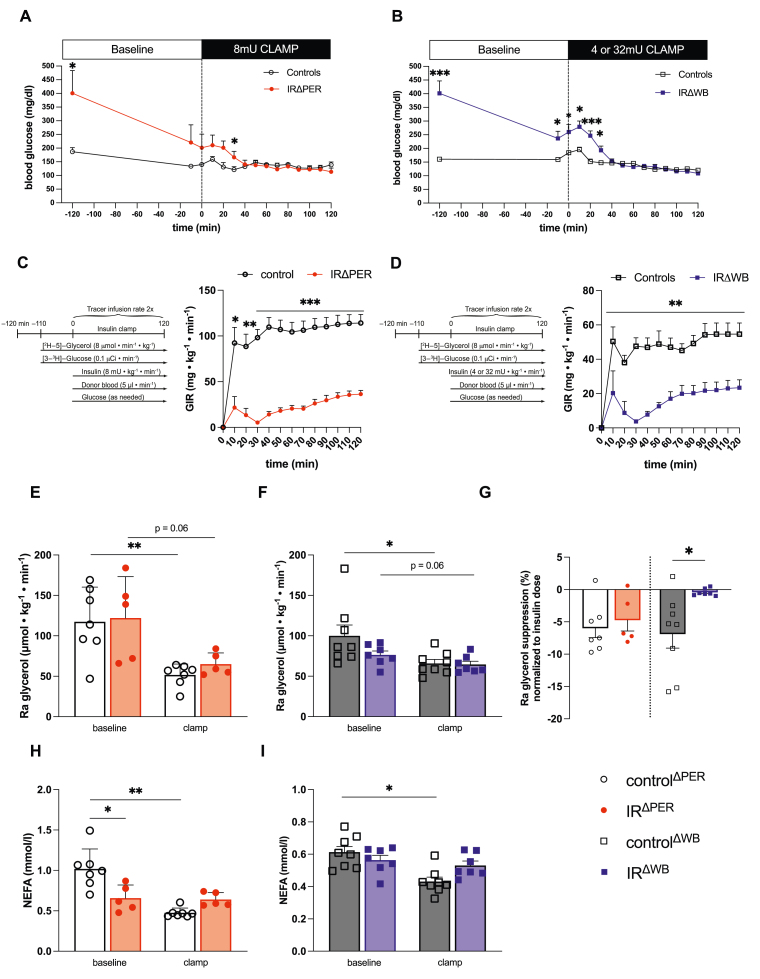
**Brain insulin receptor expression is required for the suppression of WAT lipolysis** (**A**) Blood glucose levels of IR^ΔPER^ mice and controls^ΔPER^ prior to and during an 8mU hyperinsulinemic clamp. (**B**) Blood glucose levels of IR^ΔWB^ mice prior to and during a 4mU (controls^ΔWB^) and 32mU (IR^ΔWB^) hyperinsulinemic clamp. (**C)** Schematic illustration of the hyperinsulinemic clamps and glucose infusion rates (GIR) of IR^ΔPER^ mice and controls^ΔPER^. (**D)** Schematic illustration of the hyperinsulinemic clamps and glucose infusion rates (GIR) of IR^ΔWB^ mice and controls^ΔWB^. (**E)** Rate of appearance (Ra) of glycerol of IR^ΔPER^ mice and controls^ΔPER^ at baseline and during the hyperinsulinemic clamp. (**F)** Ra glycerol of IR^ΔWB^ mice and controls^ΔWB^ at baseline and during the hyperinsulinemic clamp. (**G)** Percentage Ra glycerol suppression during the clamp in IR^ΔPER^mice, IR^ΔWB^mice and respective controls after the onset of the hyperinsulinemic clamps normalized to the insulin dose during the clamps (i.e. 8mU for IR^ΔPER^ and control^ΔPER^, 4mU for control^ΔWB^ and 32mU for IR^ΔWB^) (**H)** Plasma non-esterified fatty acids (NEFA) of IR^ΔPER^ mice and controls^ΔPER^ at baseline and during the hyperinsulinemic clamp. (**I)** Plasma NEFA of IR^ΔWB^ mice and controls^ΔWB^ at baseline and during the hyperinsulinemic clamp. Data are shown as mean ± SEM. n ≥ 5. ∗p < 0.05, ∗∗p < 0.01 ∗∗∗p < 0.001 by Student's unpaired t test (A, B, C, D, G and H) and paired t test (E, F, H and I).

To test whether insulin action in the brain is sufficient to suppress adipose tissue lipolysis, we studied two mouse models with an inducible IR knockout to define the physiological relevance of brain insulin signaling in regulating WAT lipolysis. The first mouse model lacks the IR in all tissues (IR^ΔWB^), while the second model lacks the IR in all peripheral tissues (IR^ΔPER^), but still expresses normal levels of the IR in the brain. Tamoxifen injection in adult IR^lox/lox^ Rosa26CreERT2^+/−^ mice (IR^ΔPER^) almost completely depleted IR expression in WAT and liver compared to IR^lox/lox^ Rosa26CreERT2^−/−^ mice (control^ΔPER^), while IR expression in the hypothalamus as well as in other brain regions was preserved (Figure 1A–C and Figure S1) consistent with a previous study [14]. In contrast, IR^ΔWB^ mice, which express an shRNA directed against the IR gene upon doxycycline exposure, lose IR expression in the whole body including the brain (Figure 1A–C) [14]. To define insulin action, we studied both mouse strains by hyperinsulinemic-euglycemic clamps coupled with tracer dilution techniques to track lipolysis and hGP simultaneously. As expected, both strains were insulin resistant as indicated by marked hyperglycemia and hyperinsulinemia (Figure 1D–E and Figs. S2A–B) as well as a considerably reduced glucose infusion rate (GIR) during the hyperinsulinemic clamp studies compared to the respective wild-type controls (Figure 2A–D). However, IR^ΔWB^ mice required a 4-fold higher insulin dose to achieve normoglycemia during the clamp (Figure 2A–B), demonstrating a more severe insulin resistant phenotype in IR^ΔWB^ compared to IR^ΔPER^ mice. Insulin's ability to suppress hGP and to promote glucose disposal was impaired in both IR^ΔWB^ and IR^ΔPER^ mice compared to the respective controls (Figure 2C–D and S2C-E). The rate of disappearance (Rd) revealed no significant difference between the IR depletion models and the corresponding controls (Figure S2F). However, the calculation of the Rd using the model of Steele is known to underestimate glucose disposal at high glucose turnover (especially when high insulin doses are used) [25,26]. A modified calculation of the Rd, that takes into account that the hGP can be negative, clearly indicates that the glucose uptake is impaired in both, IR^ΔPER^ and IR^ΔWB^ mice, which is in accordance with the substantially reduced GIR (Figure 2C–D and S2G). In contrast to the impaired regulation of hGP and glucose disposal, suppression of WAT lipolysis was largely preserved in IR^ΔPER^ mice, as indicated by a similar change of the rate of appearance (Ra) of glycerol during the clamp (Figure 2E,G). In contrast, insulin had only a minor effect on the Ra glycerol in IR^ΔWB^ mice compared to controls, despite substantially higher doses of insulin during the clamp (Figure 1F,G). As reported by others [14], plasma NEFAs were already reduced at baseline in IR^ΔPER^ mice and did not further decrease after induction of a hyperinsulinemic clamp (Figure 2H). Similar to the Ra glycerol, hyperinsulinemia did not suppress NEFA levels of IR^ΔWB^ mice (Figure 2I) These results support the notion that brain insulin signaling is a key determinant of WAT function in mice regulating lipolysis even in the absence of peripheral IR.

### 2.2 The hypothalamic MAPK pathway is necessary for brain insulin's anti-lipolytic action in male rats

**Figure 3:**
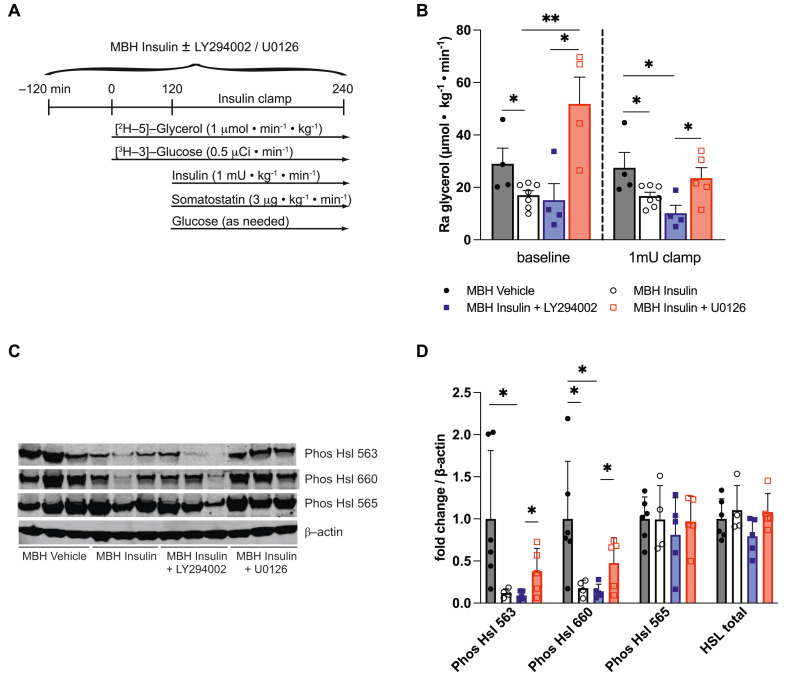
**Brain Insulin requires the MAPK signaling pathway in the MBH in order to suppress WAT lipolysis**. (**A**) Study protocol (**B**) Ra glycerol measured in plasma at baseline (TP 120) and at the end of the 1 mU · kg^−1^ · min^−1^ insulin clamp (TP 240). (**C**) Representative western blot and (**D**) quantification of HSL activation state and total expression in epidydimal WAT samples harvested at the end of a 1mU clamp. Data are shown as mean ± SEM, ∗p < 0.05, ∗∗p < 0.01 by Student's unpaired t test, n ≥ 4 per group.

Within the hypothalamus, insulin signals through the phosphatidylinositol-3-OH kinase (PI3K) and the mitogen-activated protein kinase (MAPK) pathway [21,27]. To determine the insulin signaling pathway that is required for insulin in the hypothalamus to suppress lipolysis, insulin was co–infused with either the PI3K inhibitor LY294002 or the MAPK inhibitor U0126 into the MBH of male Sprague Dawley rats and the rate of glycerol appearance was assessed using tracer dilution techniques (Figure 3A). In order to maintain circulating plasma insulin levels at basal levels (i.e. fasting condition), a 1 mU · kg^−1^ · min^−1^ pancreatic clamp was performed. MBH insulin's ability to suppress the rate of glycerol appearance was blunted by pharmacologically blocking the MAPK pathway, whereas no difference compared to the MBH insulin group was observed when the PI3K inhibitor was co-infused with MBH insulin (Figure 3B). Notably, the MAPK inhibitor abolished the antilipolytic effect of MBH insulin even before the onset of the pancreatic clamp, despite slightly higher peripheral insulin levels. Because the sympathetic nervous system and adrenergic signaling is the major driver of WAT lipolysis we assessed hormone-sensitive lipase (HSL) phosphorylation at Ser563 and Ser660, which activates HSL and is an established indicator of sympathetic nervous system outflow to WAT [28,29]. MBH insulin reduced HSL phosphorylation at serine residues 563 and 660, indicative of reduced sympathetic outflow. Co-infusion of the MAPK inhibitor, but not the PI3K inhibitor, partially prevented the activation of HSL (Figure 3C–D). Similarly, the MAPK inhibitor also partially prevented phosphorylation of a 62 kDa sized protein kinase A substrate (Figure S3), which corresponds to perilipin [9,30]. These findings corroborate the relevance of the hypothalamic MAPK pathway in regulating WAT sympathetic tone. None of the treatments altered the protein expression of adipose tissue triglyceride lipase (ATGL), HSL, perilipin, or glucose transporter 4 (GLUT4) in WAT compared to the control group (Figure S3). Taken together, these observations indicate that the MAPK pathway in the MBH contributes to the anti-lipolytic action of brain insulin signaling.

### 2.3 Impaired suppression of WAT lipolysis due to hypothalamic MAPK inhibition is associated with increased hGP in male rats

**Figure 4:**
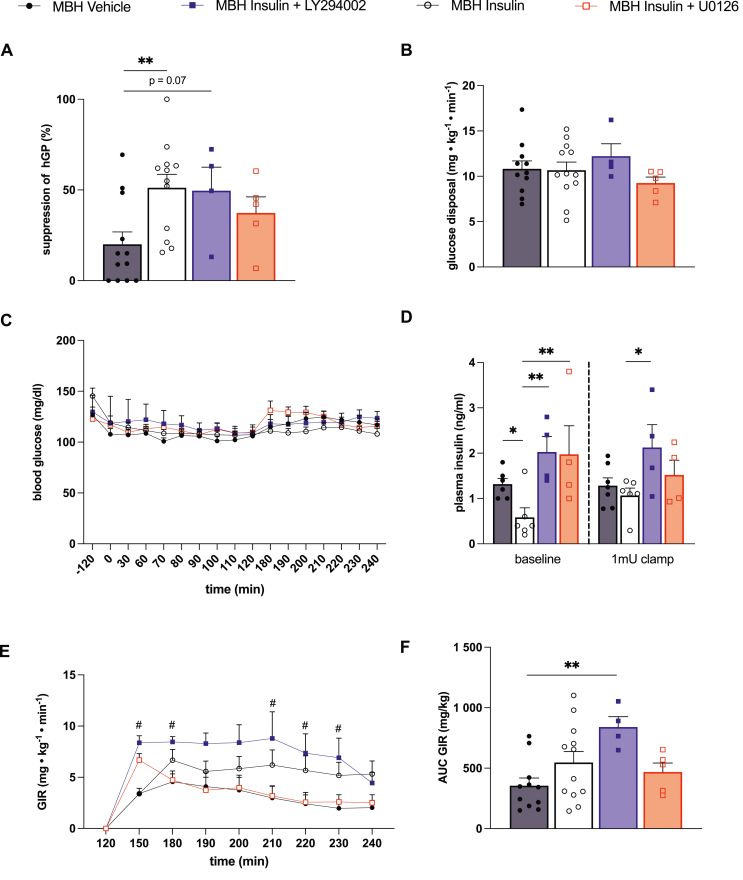
**Co–infusion of the MAP kinase inhibitor U0126 partially blocked the effects of MBH insulin on the suppression of hGP without affecting glucose disposal**. (**A**) Suppression of hGP and (**B**) glucose disposal measured with tracer dilution methodology during a 1mU · kg^−1^ · min^−1^ clamp in rats receiving an infusion of either aCSF (vehicle), insulin, insulin + PI3K inhibitor LY 294002 or insulin + MAPK inhibitor U0126 into the MBH. (**C**) Plasma insulin levels at baseline (−120 to 120 min pre–clamp period) and during the clamp (120–240 min). (**D**) Glucose levels prior to and during the clamp. (**E, F**) Glucose infusion rate (GIR) required to maintain euglycemia. Data are shown as mean ± SEM, ∗p < 0.05, ∗∗p < 0.01, ^#^p < 0.05 comparing MBH Insulin + LY294002 vs. MBH Vehicle by Student's unpaired t test; n ≥ 4 per group.

Given the role of lipolytic flux in the regulation of hGP, we next determined whether inhibition of the MAPK pathway in the MBH also affects hGP. To this end we estimated lipolysis and glucose fluxes simultaneously with tracer-dilution methodology. MBH insulin infusion suppressed hGP during the pancreatic clamp comparable to prior reports [16]. The ability of MBH insulin to suppress hGP was not substantially altered by PI3K inhibition although hGP suppression missed statistical significance compared to the control group. However, MBH insulin at least partially failed to suppress hGP when a MAPK inhibitor was co-infused, while circulating glucose and insulin levels were controlled by a pancreatic clamp (Figure 4C,D). Infusion of insulin into the MBH did not alter glucose disposal in peripheral tissues consistent with previous reports, and the inhibition of insulin signaling pathways did not alter glucose disposal (Figure 4B). The glucose infusion rate (GIR) required to maintain normoglycemia during the clamp correlated with the hGP suppression data (Figure 4A) and was higher in the MBH insulin and MBH insulin plus PI3K inhibitor groups compared to control and MBH insulin plus MAPK inhibitor (Figure 4C). The GIR during the MBH insulin plus MAPK inhibitor infusion was comparable to the control group (Figure 4E–F).

### 2.4 U0126 specifically reduced hypothalamic MAPK signaling in male rats

**Figure 5:**
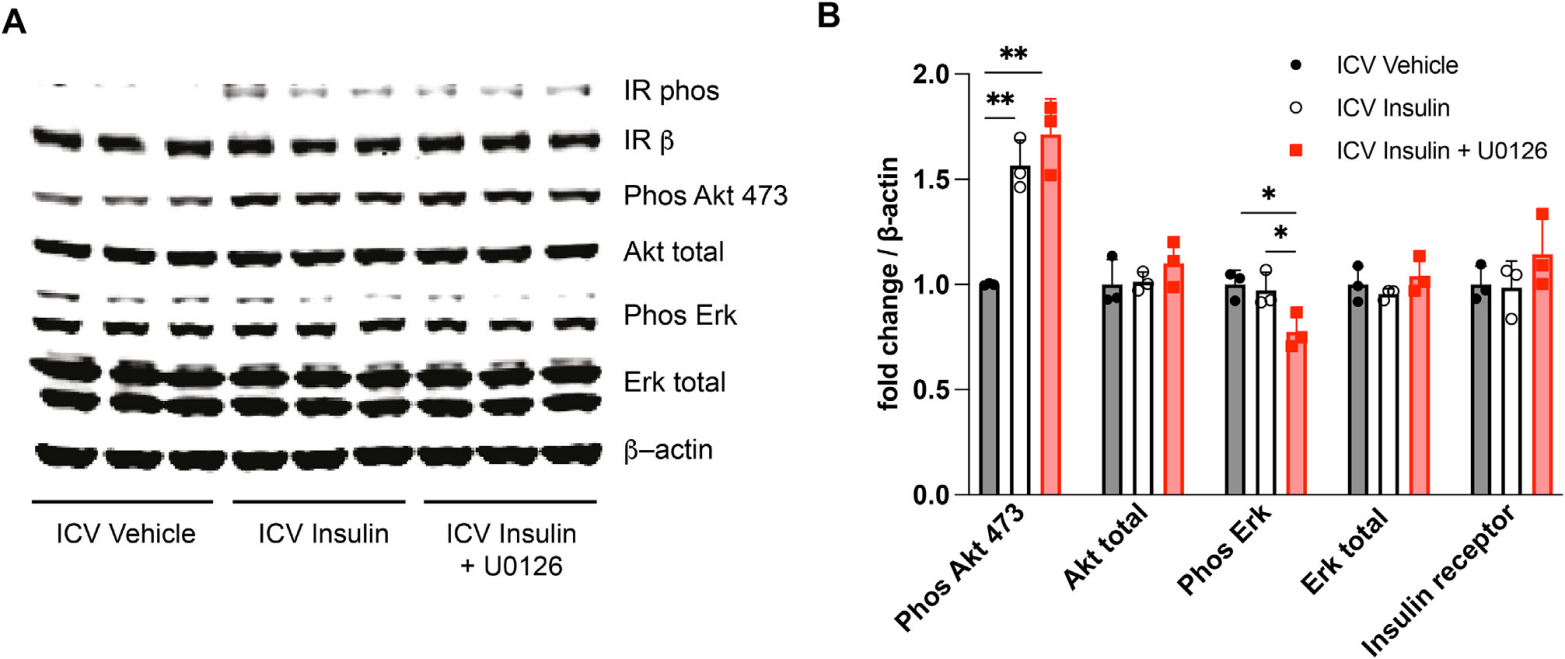
**Validation of the MAP kinase inhibitor U0126 in vivo using western blot**. (**A**) Representative western blot. (**B**) Quantification of the phosphorylation state of key components of the insulin signaling pathway. Data are shown as mean ± SEM, ∗p < 0.05, ∗∗p < 0.01 by Student's unpaired t test, n ≥ 4.

Lastly, we performed western blot analysis of the MAPK insulin-signaling pathway in the MBH. Co-infusion of insulin and U0126 reduced extracellular signal-related kinase [31] but not AKT phosphorylation confirming the specific inhibition of MAPK pathway without affecting PI3K signaling (Figure 5A,B).

## 3 Discussion

Adipose tissue insulin action, in particular its ability to suppress lipolysis, is pivotal for WAT functionality. Impaired insulin action in WAT results in unrestrained lipolysis which is a major cause of reduced lipid storage capacity in dysfunctional WAT and metabolic disease [1]. The anti-lipolytic effects of insulin are mediated both directly via IR on adipocytes and indirectly via the brain through a suppression of the sympathetic nervous system outflow to WAT [11,16]. Notably, the effects of brain insulin signaling on WAT lipolysis occur independent of adipocytes insulin action [16,32]. Here, we corroborate our previous studies [16,32] demonstrating that insulin infusion into the MBH suppresses whole body lipolysis as assessed by a stable isotope glycerol tracer. We demonstrate that insulin is still able to suppress lipolysis in mice lacking peripheral IRs as long as insulin signaling in the brain is intact emphasizing the critical role of brain insulin signaling in maintaining WAT integrity and function. Within the hypothalamus, insulin requires the MAPK pathway to regulate lipolysis, but seems to be less dependent on the PI3K pathway.

Although IR^ΔPER^ mice exhibited a similar insulin induced suppression of the Ra glycerol, the gold standard for quantifying WAT lipolysis, we observed significantly lower NEFA levels already prior to the initiation of the hyperinsulinemic clamp. ICV insulin infusion promotes fatty acid uptake in WAT via a CD36 dependent mechanism [33]. We speculate that the reduction in plasma NEFA levels is the result of increased brain insulin signaling due to the marked hyperinsulinemia observed in the IR^ΔPER^ mice compared to the controls, which then reduces sympathetic outflow to WAT and basal lipolysis, and enhances fatty acid uptake in WAT via CD36.

Brain insulin signaling not only controls adipose tissue lipolysis and lipid uptake but also participates in the regulation of hGP either directly via the parasympathetic nervous system and/or indirectly by controlling the lipolytic flux (i.e. NEFA and glycerol) to the liver, which promotes gluconeogenesis [4]. We have previously demonstrated that the suppression of WAT lipolysis after brain insulin injection correlates with the suppression of hGP [16]. Here, we studied how brain insulin action controls WAT lipolysis and hGP in two genetic and inducible loss of function mouse models. Our data informs the ongoing debate about the relative importance of brain insulin and direct hepatic insulin action in the regulation of hGP [33]. In our peripheral IR depletion model (IR^ΔPER^ mice) the suppression of hGP during a hyperinsulinemic clamp was impaired despite preserved brain insulin signaling and suppressed WAT lipolysis. In contrast, the co-injection of insulin and the MAPK inhibitor into the MBH of rats impaired both suppression of WAT lipolysis and hGP. These observations clearly indicate that both direct hepatic insulin signaling and lipolytic flux regulate hGP. The relative contribution of the two mechanisms may depend on nutritional status, with the hepatic effect predominating in the postprandial state, when insulin levels are high, whereas brain insulin signaling becomes more relevant in the fasted state. However, insulin still suppressed hGP in IR^ΔPER^ mice despite the lack of hepatic IRs (Figure S2C).

In contrast to previous studies [22,34], we saw no major changes in brain insulin's ability to suppress hGP when LY294002 was co-infused. Potential reasons for this discrepancy could be important differences in study design. In both aforementioned studies the PI3K inhibitors were given into the 3rd ventricle. Under these conditions the inhibitors likely reach a variety of additional periventricular brain regions that could affect the brain-liver interorgan crosstalk, whereas in this study only the MBH was targeted. Furthermore, we injected insulin directly into the MBH to assess hypothalamic insulin action specifically while previous studies injected insulin systemically affecting several brain regions as well as peripheral tissues. Similar to our study, but in a different brain region, inhibition of the MAPK signaling pathway, but not the PI3K pathway, within the dorsal vagal complex prevented the ability of insulin to suppress hGP in mice and rats [35]. The relevance of hypothalamic MAPK signaling in regulating systemic glucose metabolism is further supported by a recent study demonstrating that the anti-diabetic effects of FGF1 depend on intact hypothalamic ERK1/2 signaling [36]. These findings highlight that brain insulin regulates systemic glucose metabolism via multiple brain regions to control glucose homeostasis and corroborate the predominant role of the MAPK pathway in at least two brain areas in regulating glucose and lipid metabolism (i.e. the MBH and the dorsal vagal complex).

Since within the hypothalamus IRs on POMC neurons are required for brain insulin to suppress WAT lipolysis, we believe that the primary cell type responsible for the anti-lipolytic action of brain insulin signaling is of neuronal origin [18]. Whether specific blockade of MAPK signaling in POMC neurons impairs the ability of MBH insulin to suppress lipolysis remains to be tested. However, since we did not directly target neurons with our experiments, we cannot exclude that the inhibition of the MAPK pathway in other, non-neuronal hypothalamic cells could also play a role in the effects observed here. Tanycytes are specialized hypothalamic glial cells that control the transport of molecules such as leptin across the blood–brain-barrier [31,37]. The transport of leptin across the blood–brain-barrier requires intact MAPK signaling [37]. Therefore, MAPK inhibition within the MBH might have impaired the transport of other nutrient- or energy-related signals that also participate in the regulation of WAT lipolysis.

Short-term voluntary overfeeding impairs hepatic insulin sensitivity in lean men without affecting peripheral insulin action [38]. A similar response to short-term overfeeding can be observed in rats fed a high fat diet for three days. These animals develop brain insulin resistance as indicated by the failure of MBH insulin to suppress WAT lipolysis and hGP despite unaffected insulin signaling in the WAT and the liver [32]. The exact mechanisms leading to this rapid development of brain insulin resistance remain incompletely understood, but likely involves the increased expression of negative feedback inhibitors of the insulin-signaling cascade are likely involved [[39], [40], [41]]. Besides the well characterized tyrosine phosphatases PTP1B and TCPTP that dephosphorylate the IR, obese mice also exhibit an increased hypothalamic expression of the MAPK phosphatase 3 associated with a reduced phosphorylation of ERK [42]. Hence these results suggest that insufficient signaling through the MAPK pathway, as is observed in obesity, plays a role in the development of brain insulin resistance and is particularly important in the regulation of WAT insulin action.

We conclude that brain insulin signaling is pivotal in regulating WAT lipolysis explaining the relatively mild lipodystrophic phenotype in mice lacking the IR in peripheral tissue compared to an IR deficiency in the whole body [14]. Furthermore, our study identifies the MAPK pathway as essential for insulin to suppress WAT lipolysis. Conditions, in which the hypothalamic MAPK pathway is compromised, as is the case in obesity [42], can result in impaired regulation of lipolysis and WAT insulin action, leading to ectopic lipid accumulation, lipotoxicity and insulin resistance.

## 4 Methods

### 4.1 Animals

Eleven–week–old, male IR^ΔWB^ mice and 15-22–week–old male IR^ΔPER^ mice (Jackson Laboratory, Bar Harbor, Maine, USA) and their respective male control mice were housed on a 12 h light–dark cycle and fed a standard rodent diet (PicoLab Rodent Diet 20; PMI Nutrition International, St. Louis, MO, USA). Rat experiments were performed in 8–week–old, standard chow fed (Rodent Diet 5001, LabDiet, St. Louis; MO, USA), male SD rats (Charles River Breeding Laboratories, Wilmington, MA, USA), housed at ambient temperature and light controlled in separate cages. The International Animal Care and Use Committee of Mount Sinai School of Medicine, New York, NY (IACUC) approved all animal experiments.

**IR**^**ΔPER**^**mice** were generated as described [14]. Briefly, mice with a floxed insulin receptor (IR^lox/lox^) were crossed with mice that expressed a CreERT2 fusion protein under control of the Rosa26 promoter (Rosa26CreERT2; Taconic, Hudson, NY, USA; Mouse model # 6466), a tamoxifen inducible general Cre deleter. Their offspring was then crossed with IR^lox/lox^ mice to yield IR^lox/lox^Rosa26CreERT2^+/−^ mice. Tamoxifen-injected IR^lox/lox^Rosa26CreERT2^+/−^ mice were referred to as IR^ΔPER^ mice, and tamoxifen-treated IR^lox/lox^Rosa26CreERT2^−/−^ mice were used as controls (controls^ΔPER^). Genotypes were determined by PCR of tail DNA. Primer sequence: 5′-GATGTGCACCCCATGTCTG-3′, 5′-CTGAATAGCTGAGACCACAG-3′ (IR allele); 5′-ACCTGAAGATGTTCGCGATTATCT-3′, 5′-ACCGTCAGTACGTGAGATATCTT-3′ (CreERT2 transgene). To induce Cre recombination both IR^Δper^ and Control^Δper^ mice received 1.5 mg tamoxifen dissolved in 300 μl sunflower oil (both Sigma Aldrich, St. Louis, MO) via intraperitoneal injection for 5 consecutive days. Bodyweight and glucose levels were monitored daily.

**IR**^**ΔWB**^**mice**, which express an shRNA directed against the IR gene upon doxycycline exposure (described in detail in [14,43]), were purchased from Taconic, Hudson NY (Mouse model # 9189). Doxycycline-treated transgenic mice are referred to as IR^Δwb^ mice and doxycycline-treated C57BL/6Ntac mice were used as controls (Control^Δwb^ mice). IR^Δwb^ and Control^Δwb^ received drinking water supplemented with 10% sucrose and 2 mg/ml doxycycline (both Sigma Aldrich, St. Louis, MO) to induce expression of the shRNA. Drinking water was changed every 2 days. Bodyweight and glucose levels were monitored daily.

### 4.2 Mouse pancreatic clamp studies

Indwelling jugular vein catheters were implanted approximately one week prior to the clamps (Supplemental Methods). Clamp studies were performed in conscious ad libitum fed 11-22– week–old male mice. On the morning of the study, vascular lines were connected and each mouse was placed into a restrainer. The 220 min protocol consisted of a 100 min tracer equilibration followed by a 120 min clamp period. The tracer equilibration period was started at t = −100 min by infusing a 5 min 10 μCi bolus of [3-^3^H] glucose (Radiochemical concentration >97%; PerkinElmer, Waltham, MA) and a 800 μmol/kg BW bolus of [^2^H-5]-glycerol (98 atom percent excess) purchased from Isotec (Miamisburg, OH) followed by a continuous infusion of 0.1 μCi min^−1^ and 8 μmol kg^−1^ · min^−1^ infusion of [3-^3^H]-glucose and [^2^H-5]-glycerol, respectively, over the next 95 min. Before starting the insulin clamp, plasma samples were taken to determine both baseline hGP and Ra glycerol. The insulin clamp was then initiated at t = 0 min with a primed-continuous infusion of insulin (Humulin R, Lilly) consisting of a 72, 144 or 576 mU · kg^−1^ · min^−1^ bolus for 1 min followed by a continuous infusion of 4, 8 or 32 mU · kg^−1^ · min^−1^, respectively. Upon induction of the clamp the tracer infusion rate was doubled for the remainder of the experiment to reduce changes in specific activity from the equilibration period. Euglycemia was maintained by measuring blood glucose every 10 min using AlphaTrak glucose strips (Abbott, Abbott Park, IL) and infusing glucose (40% wt/vol) as necessary. Four consecutive blood samples were taken at the end of the study (t = 90, 100, 110 and 120 min) to determine hGP and Ra glycerol during the hyperinsulinemic clamp. Throughout the clamp period, mice received an infusion of saline-washed erythrocytes from donor mice at a rate of 5 μL min^−1^ to compensate for the blood loss from sampling.

### 4.3 Rat pancreatic clamp studies

Indwelling jugular vein catheters were implanted approximately one week prior to the clamps (Supplemental Methods). Rat clamp experiments were performed in conscious, non–restrained, ad libitum fed male SD rats as previously described [16]. An MBH (0.18 μl/h per side) infusion with either artificial cerebrospinal fluid (vehicle; aCSF) (Harvard Apparatus, Holliston, MA), insulin (total dose 2 μU; Humulin R, Lilly), LY294002 (total dose 0.216 nmoles; Cell Signaling Technology, Boston, MA) or U0126 (total dose 0.55 nmoles; Cell Signaling Technology, Boston, MA) was started and maintained for the entire 360 min study. After 120 min of continuous brain infusion (t = 0 min) we started a 120 min tracer equilibration period, which consisted of a 20 μCi bolus of [3-^3^H]-glucose (>97%; PerkinElmer, Waltham, MA) and a 13.3 μmol bolus of [^2^H-5]-glycerol (98%) purchased from Isotec (Miamisburg, OH) followed by a 0.5 μCi/min and 0.334 μmol/min infusion of [3-^3^H]-glucose and [^2^H-5]-glycerol, respectively. At the end of the equilibration period, arterial blood samples were taken to determine baseline GP and Ra glycerol using tracer dilution methodology. At t = 120 min an insulin clamp was started with a primed–continuous infusion of insulin (17.7 mU · kg^−1^ bolus over 1 min followed by a 1 mU · kg^−1^ · min^−1^ infusion; Humulin R, Lilly) while also infusing somatostatin (3 μg kg^−1^ · min^−1^) and continuing the tracer infusions at aforementioned rate.

### 4.4 Glucose tracer analyses

To measure plasma [3-^3^H]-glucose radioactivity samples were de–proteinated using barium hydroxide and zinc sulfate. After centrifugation the supernatant was dried overnight to eliminate tritiated water. Glucose was then redissolved in water and counted using Ultima Gold in a MicroBeta TriLux (both PerkinElmer, Waltham, MA) liquid scintillation counter. Under baseline steady state conditions the hGP equals the glucose turnover rate, which was determined from the ratio of the [3-^3^H]-glucose tracer infusion rate and the specific activity of plasma glucose. During the pancreatic clamp hGP is calculated by subtracting the glucose infusion rate (GIR) from the glucose turnover rate, which in a steady state equals the rate of glucose disposal (Rd). During high glucose turnover (especially when high insulin doses are used) the model of Steele, is known to produce negative estimates of hGP due to an underestimation of glucose disposal [25,26]. Therefore, in addition, a modified total body glucose disposal rate (modified Rd) was calculated in the mouse clamp experiments by adding the mean rate of hGP (if a positive number) to the mean glucose infusion rate (GIR) during the same period (as described elsewhere [44]).

### 4.5 Ra glycerol analyses

The plasma glycerol Ra in μmol · kg^−1^ · min^−1^ was used as an index for systemic lipolysis, as calculated by the equation

$$R_{a}=\left(\frac{{ENR}_{\inf}}{{ENR}_{pl}}-1\right)∙R$$

Where ENR_inf_ is the fractional isotopic enrichment of the infused glycerol in atom percent excess and ENR_pl_ that in the arterial plasma sample. R is the rate of the isotope infusion in μmol · kg^−1^ · min^−1^. The ^2^H-labeling of plasma glycerol was determined as follows: 20 μl of plasma was de–proteinized with 200 μl of methanol by centrifugation for 10 min at 16,100 g. The fluid fraction was then evaporated to dryness and reacted with 50 μl of bis(trimethylsilyl)trifluoroacetamide plus 10% trimethylchlorosilane for 20 min at 75 °C. Isotope enrichment was determined by gaschromatography-massspectrometry.

### 4.6 Brain signaling studies

Overnight fasted male SD rats were anaesthetized using ketamine–xylazine and then implanted with cannulae targeting the 3rd ventricle (as described in [16]). Rats were 8–weeks–old (mean bodyweight 244 g). Correct placement was verified by a food dye injection at the end of the study. Rats received two 10 μl bolus injections 15 min apart. The control group received two aCSF +5% DMSO injections, the insulin group received aCSF +5% DMSO followed by 500 mU of insulin (Humulin R, Lilly) and the MAPK inhibitor group received 5 nmoles U0126 + 5% DMSO followed by 500 mU insulin +5 nmoles U0126 + 5% DMSO. All rats were killed after anesthesia by decapitation 15 min after the last bolus and the MBH was dissected using N.I.H. style neuro punches (Fine Science Tools, Foster City, CA) and snap frozen in liquid nitrogen. MBH was dissected as follows: The rat brain was transferred to an acrylic brain matrix (Stoelting, Wood Dale, IL) with sagittal sections at 1 mm intervals. We then obtained a 1 mm sagittal brain slice according to the coordinates in the Paxinos Rat Brain Atlas (bregma −3.24) [45] by cutting the brain with a razor blade, which was then transferred to a microscope glass slide and snap frozen in liquid nitrogen for a few seconds. We then dissected the MBH using N.I.H. style neuro punches (Fine Science Tools, Foster City, CA) with a 0.75 mm diameter and snap froze the biopsies in liquid nitrogen for western blot analyses.

### 4.7 Western blot analyses

The confirmatory western blot in Figure 1A was performed in an independent cohort of mice prior to the clamp. Tissues for the western blots in Figure 1B were harvested immediately after the clamps. Frozen tissue samples were homogenized in 20 mM MOPS, 2 mM EGTA, 5 mM EDTA, 30 mM sodium fluoride, 40 mM beta-glycerophosphate, 10 mM sodium pyrophosphate, 2 mM sodium orthovanadate, 0.5% NP-40 and complete protease inhibitor cocktail (Roche, Nutley, NJ) and centrifuged at 13,000 g for 20 min at +3 °C (WAT: 3 °C). The supernatant was then collected carefully avoiding the lipid layer on top when processing WAT. Protein concentration was measured with a BCA protein quantification kit (Thermo Scientific, Waltham, MA). Protein extracts were separated on 4–12% NuPAGE gels (Invitrogen, Carlsbad, CA) and blotted onto Immobilon FL PVDF (Millipore, Billerica, MA). Membranes were blocked at room temperature for 1 h in Odyssey LI-COR Blocking Buffer (LI-COR, Lincoln, NE) 1:1 diluted in TBS and incubated in primary antibodies in 1:1 Blocking Buffer/TBS-T overnight at 4 °C. Primary antibodies against IRβ (sc-57342, Santa Cruz Biotechnology, Santa Cruz, CA), Glut 4 (ab654, Abcam, Cambridge UK), Akt (#9272), phos Akt (#4060), Erk (#9102), phos Erk (#9101), Hsl (#4107), phos Hsl 563 (#4136), phos Hsl 660 (#45804), Hsl 565 (#4137), Atgl (#2138), phospho-PKA substrate antibody (#9621) were used (all Cell Signaling Technology, Beverly MA if not stated otherwise). Andrew Greenberg kindly provided the perilipin antibody (Souza et al. J Biol Chem 2002). After three consecutive 5 min washes in TBS-T (0.1%), blots were incubated with Dylight 680-conjugated goat anti–rabbit IgG and Dylight 800-conjugated goat anti–mouse IgG (both Thermo Scientific, Waltham, MA) for 1 h at room temperature in blocking buffer containing 0.1% TBS-T and 0.1% SDS. After three washes in TBS-T and a final wash in TBS, the blots were scanned with the LI-COR Odyssey (LI-COR, Lincoln, NE) and quantified with Odyssey 3.0 software based on direct fluorescence measurement.

### 4.8 Statistics

All values are presented as a mean ± SEM. Data were analyzed using GraphPad Prism 9 for macOS Version 9.1.2 (GraphPad Software, San Diego, California USA (www.graphpad.com). Comparisons within groups were made using a paired two-tailed Student's t test. Between-group differences were compared with an unpaired two-tailed Student's t test. Differences were considered statistically significant at p < 0.05.

## Authors contributions

Conceptualization: T.S., C.B; Methodology: C.B., T.S.; Formal analysis: M.M., T.S., C.B.; Investigation: T.S., K.S., B.C., J.O., M.P.; Resources: C.B.; Writing – Original Draft: M.M., Writing – Review & Editing: T.S., C.B.; Visualization: M.M., T.S.; Supervision: C.B.

## Declaration of competing interest

The authors declare the following financial interests/personal relationships which may be considered as potential competing interests: CB received research support from Pfizer and consulted for Boehringer and Novo Nordisk.

## Data Availability

Data will be made available on request.
