# Supplementary figures and images for: Brain insulin signaling suppresses lipolysis in the absence of peripheral insulin receptors and requires the MAPK pathway
Source: Mol Metab. 2023 Apr 24;73:101723. doi: 10.1016/j.molmet.2023.101723 (PMC10193009; doi:10.1016/j.molmet.2023.101723)

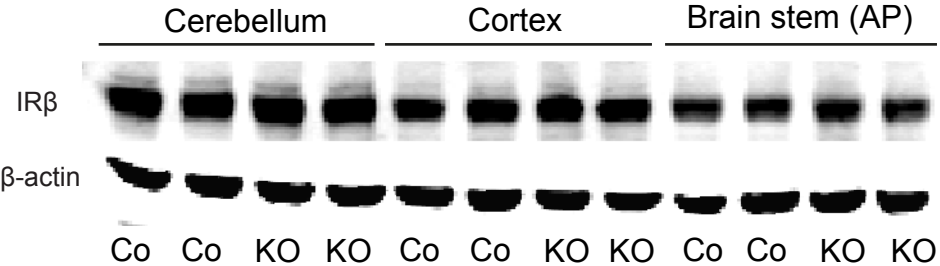

Supplement: Figure S1 [file mmc1.pdf]

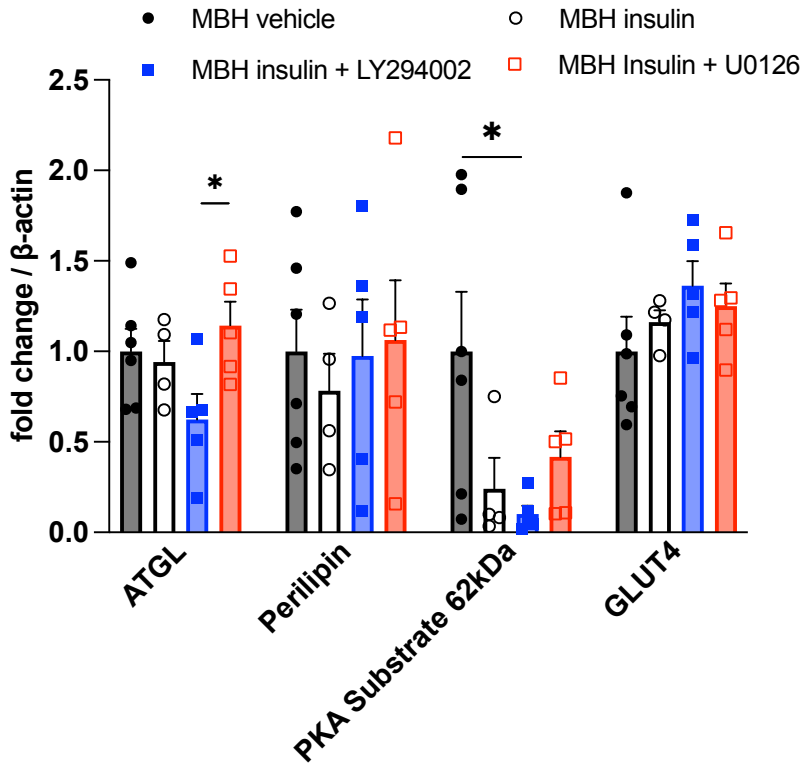

Supplement: Figure S3 [file mmc3.pdf]
