# Supplementary material for: Brain insulin signaling suppresses lipolysis in the absence of peripheral insulin receptors and requires the MAPK pathway
Source: Mol Metab. 2023 Apr 24;73:101723. doi: 10.1016/j.molmet.2023.101723 (PMC10193009; doi:10.1016/j.molmet.2023.101723)
Supplement: Figure S2 [file mmc2.pdf]

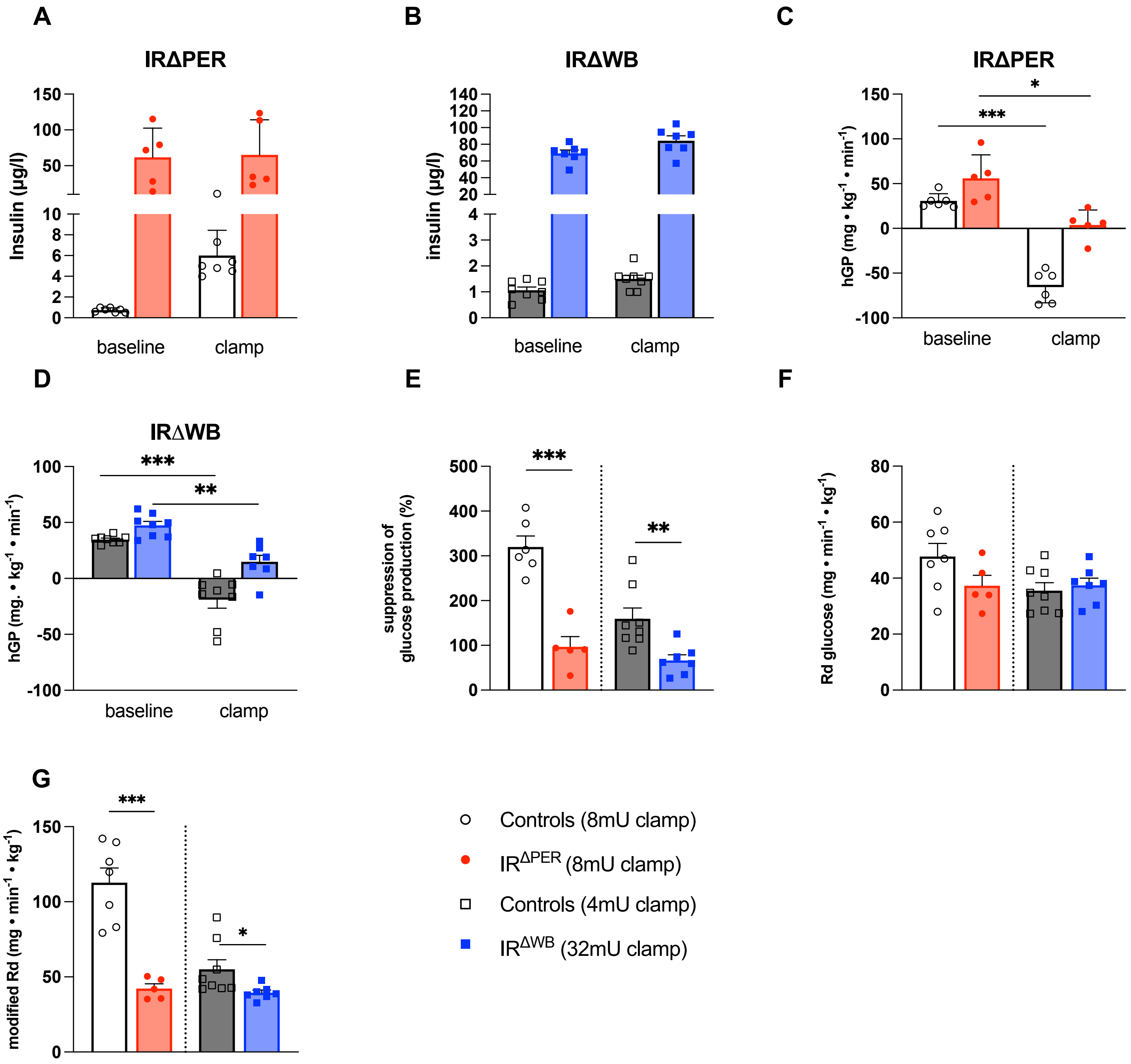

A
IRΔPER
Insulin (µg/l)
150
100
50
10
8
6
4
2
0
baseline
clamp
B
IRΔWB
insulin (µg/l)
140
120
100
80
60
40
20
4
3
2
1
0
baseline
clamp
C
IRΔPER
hGP (mg • kg-1 • min-1)
*
***
150
100
50
0
-50
-100
baseline
clamp
D
IRΔWB
hGP (mg. • kg-1 • min-1)
***
**
100
50
0
-50
-100
baseline
clamp
E
suspection of glucose production (%)
***
**
500
400
300
200
100
0
F
Rd glucose (mg • min-1 • kg-1)
80
60
40
20
0
G
modified Rd (mg • min-1 • kg-1)
***
*
150
100
50
0
Controls (8mU clamp)
IRΔPER (8mU clamp)
Controls (4mU clamp)
IRΔWB (32mU clamp)
